# Don't Miss the Forest for the Trees: How Abstracting Nature Can Get Us Closer to Our Goals

**DOI:** 10.1111/gcb.70146

**Published:** 2025-04-04

**Authors:** Jake Lawlor

**Affiliations:** ^1^ Department of Biology McGill University Montreal Quebec Canada

How will natural environments change in the future? As climate envelopes shift across Earth's surface (Burrows et al. 2011) and species redistribute across the globe to follow (Pecl et al. 2017), predicting ecological outcomes is crucial for guiding intervention, management, and adaptation strategies for biodiversity changes. However, the scale and ecological resolution with which we assess biodiversity changes can greatly influence both the methods that we choose and the insights that we glean.

Understanding complex natural ecosystems and their responses to climate change sometimes requires abstraction—condensing primary data to extract broad‐scale patterns while necessarily sacrificing some details. For example, abstracting species occurrences to richness, or species interactions to network links can reveal patterns about the structure and connectance of ecosystems. However, this process comes with a tradeoff, because gaining these broad‐scale insights generally means losing information about the exact species or events driving these patterns. Abstractions into functional groups, genotypes, or community‐level attributes are especially useful for assessing ecological responses to climate change (Pereira et al. 2013), and projecting these metrics into the future can provide critical insights into how ecosystems might differ under new climate conditions. Typically, projections of community‐level variables are built by first modeling individual species' responses to future climates, then summarizing species‐level predictions to higher levels. However, in many cases, community‐level responses can instead be predicted directly (Nieto‐Lugilde et al. 2017).

In a recent study published in Global Change Biology, Gougherty et al. (2024) demonstrate the latter approach. Their study examines how an abstracted community‐level variable—community composition—might change in response to changing climates in forest communities across North America. They use an extensive dataset of tree distributions paired with multiple contemporary climate parameters to calibrate a generalized dissimilarity model (GDM, Ferrier et al. 2007) that predicts the magnitude of compositional dissimilarity between any two forest communities (20 km raster cells) as a function of the climatic distance between them. They then apply their model to end‐of‐century climate projections to predict magnitudes of compositional dissimilarity between present and future forests without projecting the compositions of future forests themselves. In other words, they model changes to the forests without modeling changes to the trees. By shifting the focus of their question from “how might communities differ in future climates?” to “how different might communities in future climates be?”, their approach targets ecological responses to climate change from an abstracted lens.

Gougherty et al. identify broad‐scale patterns of compositional dissimilarity between present and future forests, which they summarize in three ways. First, they quantify potential magnitudes of compositional change at the local scale by predicting compositional dissimilarity between each forested cell in the present and itself in the future. Then, they adapt methods from climatology (sensu Williams et al. 2007) to identify regions of potentially disappearing and novel forest communities under climate change: present communities with no suitably‐similar future analogs across the continent, and future communities with no suitably‐similar present analogs across the continent, respectively. Their study presents regional patterns of climate risk by each of these metrics, but also raises the question: What advantages does modeling community change directly provide over analyzing the same data with a more traditional species‐centric workflow?

Directly modeling community‐level changes streamlines ecological projections. While a species‐centric workflow could arrive at similar community‐level insights, the process would involve some extra steps. Projecting local compositional change and identifying novel and disappearing communities with a species distribution model (SDM) workflow would require fitting and projecting models for each species (over 300, in this case)—or even multiple models per species to ensemble outputs—all before computing cell‐to‐cell comparisons to quantify magnitudes of compositional dissimilarity between sites. Gougherty et al. bypass these steps by modeling community change directly, with compositional dissimilarity as the response variable of their model. Although computational limitations in ecology are easing with the rise of data availability and integrations like artificial intelligence, the speed and directness of their approach is valuable, especially given the urgency of understanding climate risks to biodiversity for global conservation.

Direct community‐level insights can align with emerging conservation priorities. As management strategies increasingly integrate community‐level metrics alongside single‐species plans, direct approaches for modeling community‐level changes can offer rapid insights to practitioners. For example, the Group on Earth Observations Biodiversity Observation Network (GEO BON) has identified numerous Essential Biodiversity Variables for the focus of biological monitoring in the Anthropocene, of which community composition is one of six classes (Pereira et al. 2013). Gougherty et al.'s local change analysis demonstrates how magnitudes of community compositional change can be predicted at large scales directly, potentially informing efforts for accurately monitoring these changes. Because confidently detecting changes that are subtle requires higher sampling effort than detecting changes that are large, regional‐to‐continental scale predictions of local compositional change could help guide resource allocation for monitoring schemes. Moreover, their analysis of novel and disappearing ecological communities could provide a framework for assessing climate risks within management areas. In North America, novel and disappearing climate conditions are already evaluated within protected area networks for their presumed downstream impacts on biodiversity (Batllori et al. 2017). Incorporating projections of shifting, novel, and disappearing ecological communities within the same zones could add a new perspective on climate risks and needs for intervention within conservation networks.

Community‐level models can also benefit from the simplicity of their outputs. While species‐centric and community‐level approaches for projecting compositional change both ultimately assume that species distributions will shift with climate change, community‐level models might avoid some of the compounded uncertainties associated with stacking multiple species‐level predictions. For example, species‐centric workflows often rely on arbitrary thresholds to transform projected habitat suitability values into species presence, which can inflate estimates of species richness across space (Deschamps et al. 2023). In a similar vein, correlative SDMs often exclude biotic interactions, meaning that species with similar climate niches but strong competitive exclusion could end up with identical projected distributions in future climates (Nieto‐Lugilde et al. 2017). By abstracting to the community level from the start, Gougherty et al.'s approach sidesteps some of these issues; biotic interactions are implicitly captured through co‐occurrence patterns in the input data (Nieto‐Lugilde et al. 2017), and the model response variable portrays potential changes to the community without wagering on the fates of individual species. While community‐level models of course come with uncertainties and assumptions of their own (Ferrier et al. 2007; Nieto‐Lugilde et al. 2017), they avoid compounding error from stacking tens‐to‐hundreds of individual species‐level models.

Despite the benefits of modeling abstracted ecological variables directly, the associated loss of detail can limit the actionability of results—if we expect changes in the forests, what do we do about the trees? Gougherty et al.'s analysis reveals patterns of potential ecological change at a broad scale, but leaves some areas for improvement, which authors discuss in their article. First, modeling coarse community‐level variables omits context that lower levels could provide; predictions of compositional change alone cannot guide interventions for individual species, and identical compositional change values can represent very different ecological realities given differences in species richness. When these additional levels of resolution are useful, linking community dissimilarity models to kernel regression procedures in order to provide species‐level outputs (Ferrier et al. 2007), or pairing community composition models with additional macroecological models that predict other attributes such as richness could fill some of these gaps. Second, predicted changes in community compositions do not reveal how these changes could affect species, services, or people. Integrating trait‐based approaches into community‐level models could increase the interpretability of results by identifying how ecological functions could shift, emerge, or disappear in future climates (Gougherty et al. 2024; Nieto‐Lugilde et al. 2017). Third, identifying communities that *could* change is not a guarantee that they *will*, so just like with a species‐level approach, validation of predictions will be a crucial step. Some earlier applications of compositional GDMs have made predictions of community change at timescales that are now on the horizon (as early as 2030, e.g., Ferrier et al. 2012), providing potential opportunities to assess the extent to which modeled compositional changes are realized in empirical measurements. Finally, comparing community‐level predictions made from direct approaches to those made from species‐centric approaches could yield valuable insights. If results align, approaches could be interchangeable based on the limitations of the input data or the specifics of the research question. If they differ, further investigation would be necessary to determine which method better captures ecological realities, and where and why the differences arise.

Ultimately, strategies for anticipating ecological responses to climate change reflect philosophical and practical questions about which aspects of nature we prioritize. If conservation efforts prioritize outcomes for individual species of economic, environmental, or cultural importance, then modeling changes at a species level is necessary. However, if conservation efforts prioritize higher‐level metrics, Gougherty et al. demonstrate a potentially powerful—although to be further tested—alternative. By integrating approaches and understanding the benefits and tradeoffs of each, we can better anticipate and respond to the challenges of biodiversity loss in a rapidly changing world.

## Conflicts of Interest

The author declares no conflicts of interest.

## Linked Article

This article is a Invited Commentary on Gougherty et al., https://doi.org/10.1111/gcb.17605.

## Data Availability

The author has nothing to report.
